# Identification of acetyltransferase genes (*HAT1* and *KAT8*) regulating HBV replication by RNAi screening

**DOI:** 10.1186/s13578-015-0059-1

**Published:** 2015-12-04

**Authors:** Hui Wang, KeHui Liu, Bernard A. M. Fang, HaiQing Wu, FengDi Li, XiaoGang Xiang, WeiLiang Tang, GangDe Zhao, LanYi Lin, Shisan Bao, Qing Xie

**Affiliations:** Department of Infectious Diseases, Ruijin Hospital, School of Medicine, Shanghai Jiao Tong University, Shanghai, China; Discipline of Pathology, School of Medical Sciences and The Bosch Institute, Charles Perkins Centre, The University of Sydney, Sydney, NSW 2006 Australia; Central Clinical School, Sydney Medical School, The University of Sydney, Sydney, NSW 2006 Australia; Department of Cardiology, Shanghai First People’s Hospital, School of Medicine, Shanghai Jiao Tong University, Shanghai, China

**Keywords:** *KAT8*, *HAT1*, HBV replication, pgRNA, RNAi screening

## Abstract

**Background:**

The initiation of hepatitis B virus (HBV) replication involves the formation of covalently closed circular DNA (cccDNA) and its transcription into pregenomic RNA (pgRNA) in hepatocyte nuclei. The regulatory mechanism of HBV replication by acetyltransferase is thus far not well understood, but human acetyltransferase has been reported as being involved in the regulation of HBV replication.

**Results:**

Depletion of *KAT8* or *HAT1* via RNA interference (RNAi) markedly down-regulated HBV-DNA and pgRNA levels in HepG2.2.15 cells, with *KAT8* knockdown reducing both HBsAg and HBeAg more than *HAT1* knockdown. Consistent with these observations, HBV replication regulators hepatocyte nuclear factor-4-α (HNF4α) and peroxisome proliferator-activated receptor gamma coactivator- (PPARGC-) 1-α were decreased following knockdown of *HAT1* or *KAT8*.

**Conclusions:**

These data suggest that *KAT8* or *HAT1* regulate HBV replication and may be potential drug targets of anti-HBV therapy.

**Electronic supplementary material:**

The online version of this article (doi:10.1186/s13578-015-0059-1) contains supplementary material, which is available to authorized users.

## Background

Chronic hepatitis B virus (HBV) infection is a major global health issue, with over 350 million infected patients, who are subsequently susceptible to development of cirrhosis and hepatocellular carcinoma (HCC) worldwide [1, 2]. HBV is a noncytopathic virus with a 3.2-kb, partially double-stranded, relaxed circular (RC) DNA genome [3]. Following HBV infection, RC DNA translocates into nucleus, where it is repaired by cellular polymerase to form covalently closed circular DNA (cccDNA) that is subsequently transcribed into pre-core RNA, pgRNA, surface mRNA and X mRNA [4]. Viral RNAs are then transported to cytoplasm for translation into viral proteins (e.g., core proteins that are assembled to capsids). The newly synthesized viral genome can either be enveloped into mature viral capsids for secretion or re-enter the nucleus to replenish the cccDNA pool [3]. Residual HBV DNA reappears in serum and viral capsids later [5] but can be cleared to undetectable levels following treatment. In light of this, chronic HBV infection is thus largely attributed to persistence and replication of HBV cccDNA.

Nuclear HBV cccDNA is organized into a mini-chromosome by both histone and nonhistone proteins and is regulated in a manner similar to cellular chromatin [6]. A modification of chromatin architecture is required to allow access of condensed genomic DNA by the regulatory transcription machinery protein. Covalent histone modifications can be performed by enzymes such as histone acetyltransferases (HATs) [7]. Furthermore, multiple studies have observed that inhibition of histone deacetylase (HDAC), trichostatin A (TSA), valproic acid (VPA) and nicotinamide (NAM) enhances HBV replication, suggesting that histone acetylation may promote HBV replication [7, 8]. Taken together, this suggests that epigenetic regulation of cccDNA mini-chromosome is implicated in the early events of HBV replication [6, 7].

To investigate the acetyltransferases implicated in HBV replication, RNA interference screening was performed using a lentiviral shRNA library. By comparing the levels of hepatitis B surface antigen (HBsAg) and pgRNA, depletion of histone acetyltransferase 1 (*HAT1*) or lysine acetyltransferase 8 (*KAT8*) markedly repressed HBV replication. In particular, knockdown of *KAT8* by lentivirus-mediated shRNA resulted in decreased HBsAg and hepatitis B envelope antigen (HBeAg) levels. In addition to this, knockdown of *HAT1* or *KAT8* down-regulated the expression of hepatocyte nuclear factor-4-α (HNF4A) and peroxisome proliferator-activated receptor gamma coactivator- (PPARGC-) 1-α at the transcriptional level. These observations suggest that both HAT1 and KAT8 may regulate HBV replication.

## Results

### RNAi screening reveals acetyltransferases regulate HBV replication

HepG2.2.15, a stable cell line in which complete HBV-DNA is integrated into the cell genome, can constitutively express HBV genes and release HBV capsids, as reflected by the high levels of HBsAg and HBeAg detected in conditioned culture supernatant [9]. In light of these characteristics, HepG2.2.15 is thus an ideal cell model to study HBV replication. As HBsAg is an abundant viral surface antigen of HBV and can be detected at an appropriate sensitivity in the sera of HBV carriers [10], HBsAg was used as the marker for the primary RNAi screen. *KAT5* shRNA lentivirus served as a positive control, as it has been reported that KAT5 binds to HBV cccDNA and regulates HBV replication [7], whereas the lentiviral vector served as a negative control.

**Fig. 1:**
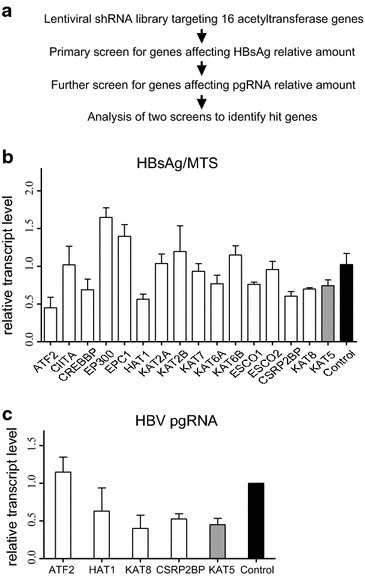
RNAi screening reveals acetyltransferases regulate HBV replication. **a** Flowchart of RNAi screening. **b** The relative HBsAg after screen. HepG2.2.15 cells were infected with lentiviral shRNA library. HBsAg was measured by ELISA and cell growth was determined by MTS assay at 96 h after infection. The relative HBV level is given by the ratio of HBsAg/MTS. **c** Relative levels of pgRNA after screen. pgRNA was measured by qRT-PCR at 72 h after infection. The lentiviral vector served as a negative control and, *KAT5* shRNA lentivirus served as a positive control, as it was reported that *KAT5* bound to HBV cccDNA and regulate HBV replication

The HBsAg level in cell supernatant was measured by ELISA, and cell viability was analyzed by cell proliferation assay at 96 h after lentiviral infection. *Via* subsequent analysis of the ratio of HBsAg to the number of cells, RNAi depletions of *ATF2*, *HAT1*, *CSRP2BP*, and *KAT8* decreased the level of HBV in the supernatant, with a similar pattern was observed in *KAT5* (Fig. 1b). To further verify the roles of these target genes in HBV replication, we investigated the relative pgRNA level after their knockdown, using qRT-PCR, as pgRNA not only serves the template of HBV RC DNA but also encodes viral replication proteins (i.e., the polymerase (P) and core protein (C)) [11, 12]. We observed RNAi depletion of *HAT1* or *KAT8* resulted in a markedly decrease by ~40 and ~60 % in the level of pgRNA, which is consistent with the level observed in *KAT5* [7], whereas *ATF2* knockdown did not show this trend (Fig. 1c). Taken together, HAT1 and KAT8 are suggested to be involved in the regulation of HBV replication.

### HAT1 and KAT8 regulate HBV replication by suppressing the expression of HBsAg, HBeAg and HBV-DNA

**Fig. 2:**
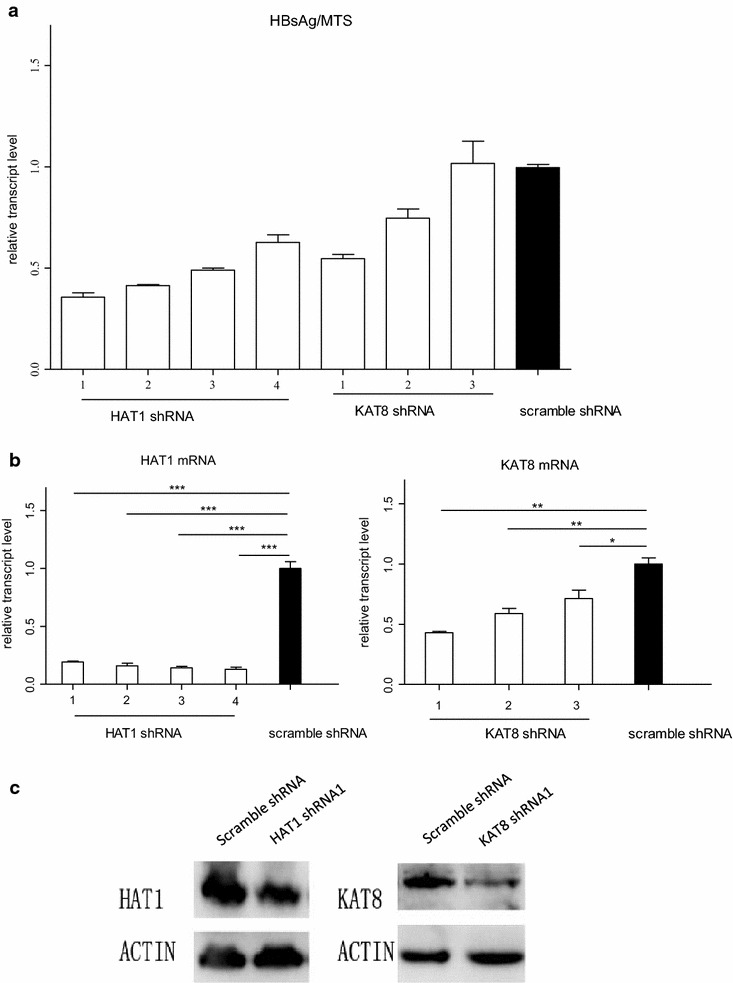
The most inhibitory shRNAs of each of HAT1 or KAT8 in HepG2.2.5 cells. **a** Relative levels of HBsAg in HepG2.2.15 cells at 96 h after infection with lentiviral shRNAs of hit gene *HAT1* and *KAT8*. The lentiviral vector served as a negative control. **b** Relative levels of *HAT1* and *KAT8* mRNA in HepG2.2.15 cells at 72 h after infection with shRNA lentivirus. Scrambled shRNA served as a negative control. (*P < 0.05, **P < 0.001). **c** Protein level of HAT1 and KAT8 in HepG2.2.15 cells after infection with most inhibitory lentiviral shRNAs. β-actin served as an internal control

The modulatory role of each shRNAs for both *HAT1* and *KAT8* in HBV replication was determined using a lentiviral vector as a carrier (Fig. 2a). *HAT1* shRNA1 inhibited HBV replication by 0.64 (SD: 0.02) folds. *HAT1* shRNA2 inhibited HBV replication by 0.59 (SD: 0.01) folds. *HAT1* shRNA3 inhibited HBV replication by 0.51 (SD: 0.01) folds. *HAT1* shRNA4 inhibited HBV replication by 0.37 (SD: 0.04) folds. *KAT8* shRNA1 inhibited HBV replication by 0.45 (SD: 0.02) folds. *KAT8* shRNA2 inhibited HBV replication by 0.25 (SD: 0.05) folds. *KAT8* shRNA3 promoted HBV replication by 0.02 (SD: 0.11) folds but this observation was not statistically significant (*P* > 0.05). Other than *KAT8* shRNA3, all of the lentiviral shRNAs had a statistically significant inhibitory effect on HBV replication.

Furthermore, we also detected the inhibition effect of each shRNAs for both *HAT1* and *KAT8*. It turned out that each four shRNAs for *HAT1* effectively reduced HAT1 expression level (*P* < 0.001 for all). For shRNA1 and shRNA2 of *KAT8*, there was a significant knockdown effect (*P* < 0.01, respectively). *KAT8* shRNA3 restrained KAT8 replication by 0.29 (SD: 0.07) folds and this observation also had a statistically significant inhibition (*P* < 0.05). The result above was shown that each shRNAs for KAT8 effectively reduced KAT8 levels. However, only 2 of them resulted in a lower of HBsAg whereas the third one had no effect. As a result, it might indicate that inhibition of KAT8 may not have an impact on HBsAg level.

After that, we tested the strongly inhibitory shRNA of each of *HAT1* or *KAT8* in HepG2.2.5 cells, *HAT1* shRNA1 and *KAT8* shRNA1, respectively. There was strong evidence suggesting that both *HAT1* shRNA1 and *KAT8* shRNA1 had a significant knockdown effect (*P* < 0.001 and *P* < 0.01, respectively), as observed via qRT-PCR and Western blot (Fig. 2b, c).

**Fig. 3:**
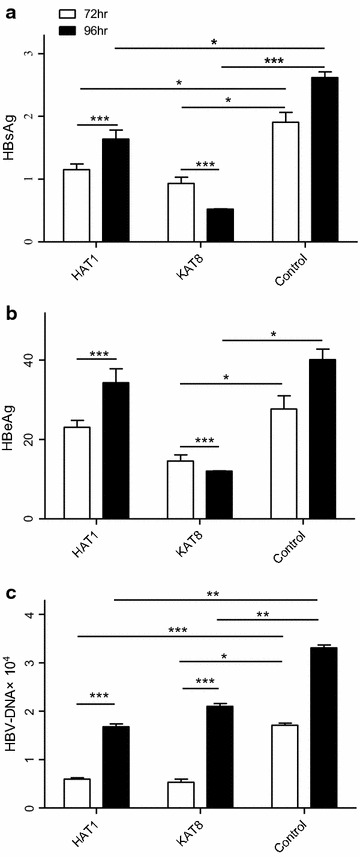
HAT1 and KAT8 regulate HBV replication by suppressing the expression of HBsAg, HBeAg and HBV-DNA. **a** Relative levels of HBsAg at 72 and 96 h after infection with lentiviral shRNAs. **b** Relative levels of HBeAg at 72 and 96 h after infection with lentiviral shRNAs. **c** Relative levels of HBV-DNA at 72 and 96 h after infection with lentiviral shRNAs. (*P < 0.05, **P < 0.001, ***P < 0.0001)

**Fig. 4:**
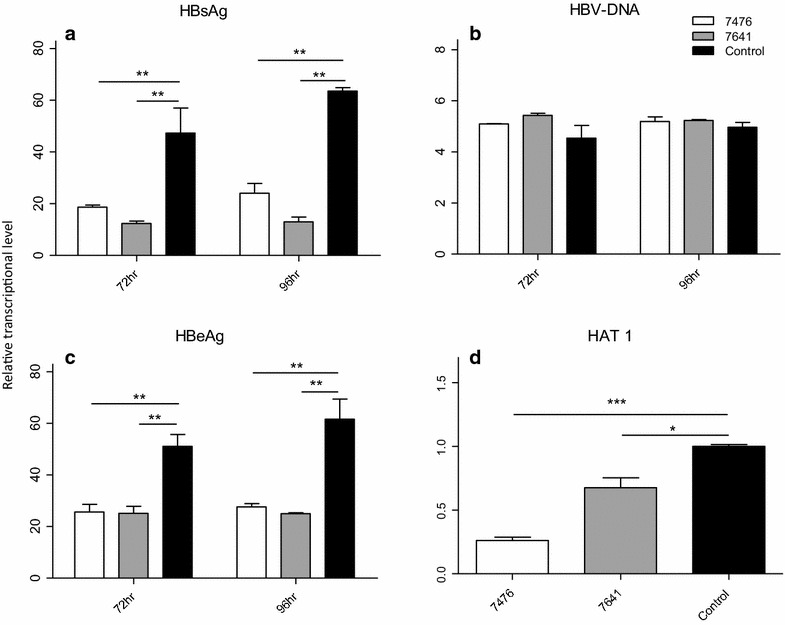
The inhibition of each of HAT inhibitors on HBV replication or HAT1 expression. **a** Relative levels of HBsAg at 72 and 96 h after treatment of HAT inhibitors. **b** Relative levels of HBV-DNA at 72 and 96 h after treatment of HAT inhibitors. **c** Relative levels of HBeAg at 72 and 96 h after treatment of HAT inhibitors. **d** Relative levels of *HAT1* mRNA in HepG2.2.15 cells at 72 h after treatment of HAT inhibitors. (*P < 0.05, **P < 0.001, ***P < 0.0001)

The levels of HBsAg, HBeAg, and HBV-DNA at 72 and 96 h after shRNA transfection were determined. The expression of HBsAg was suppressed in these three samples at 72 or 96 h, compared to control following *HAT1* or *KAT8* shRNA treatment (Fig. 3a). HBeAg was suppressed following *KAT8* shRNA treatment only (Fig. 3b). As the duration of RNAi treatment progressed, the suppression of HBsAg or HBeAg expression was enhanced in all three samples after *KAT8* shRNA treatment (both *P* < 0.0001). In contrast, up-regulation of HBsAg or HBeAg was observed between 72 and 96 h after *HAT1* shRNA treatment (both *P* < 0.0001). Furthermore, HBV-DNA was also inhibited by *HAT1* or *KAT8* shRNAs treatment (Fig. 3c), but less inhibition of HBV-DNA was observed at 96 h, compared to at 72 h, after *HAT1* or *KAT8* shRNA treatment (both *P* < 0.0001). Moreover, the levels of HBsAg, HBeAg, and HBV-DNA were determined at 72 and 96 h after *HAT1* inhibitor. The expression of HBsAg (Fig. 4a) and HBeAg (Fig. 4b) were suppressed by inhibitor 7476 (*P* < 0.001) or 7641 (*P* < 0.001) at both 72 and 96 h compared to control. No significant difference of HBV-DNA was observed following such treatments (Fig. 4c). There was significant suppression of *HAT1* following inhibitor 7476 (*P* < 0.05) or 7641 (*P* < 0.0001) (Fig. 4d). It was demonstrated that the effects of HAT1 shRNAs and HAT1 inhibitors were similar but not exactly the same. The inhibitor potently reduced HBsAg throughout the 96 h trial but not the same as HAT1 shRNAs (Figs. 3a, 4a), and HBV-DNA was inhibited by HAT1 shRNAs treatment (Fig. 4b) but there was no significant difference of HBV-DNA following inhibitor treatment (Fig. 3c).

### HAT1 and KAT8 regulate the transcription level of *HNF4A* and *PPARGC*

To investigate the mechanism by which *HAT1* or *KAT8* affected HBV replication, the expressions of HBV replication-related transcription factors *Foxa1*, *Foxa2*, *Foxa3*, *CEBP*, *HNF1A*, *HNF1B*, *HNF4A,PPAR*, *FXR*, *FTF*, and *PPARGC* were detected using qRT-PCR analysis.

**Fig. 5:**
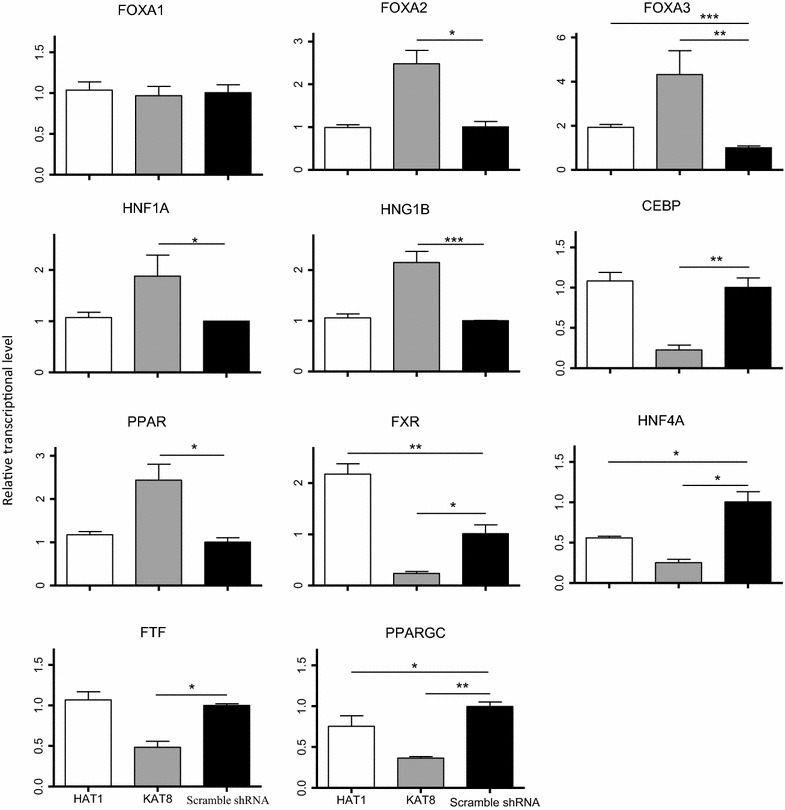
Relative levels of HBV replication regulators at 72 h after lentiviral infection. Scrambled shRNA was used as a negative control (*P < 0.05, **P < 0.001, ***P < 0.0001)

The transcription levels of *HNF4A* were found to be suppressed by 0.44 (SD: 0.02) folds and 0.75 (0.04) folds in HepG2.2.15 cells infected with *HAT1* shRNA and *KAT8* shRNA, respectively (Fig. 5). The transcription levels of *PPARGC* were found to be suppressed by 0.25 (SD: 0.13) folds and 0.64 (SD: 0.02) folds in HepG2.2.15 cells infected with *HAT1* shRNA and *KAT8* shRNA, respectively (Fig. 5). This suggests that the acetyltransferases (HAT1 or KAT8) might regulate HBV replication via the transcription factor HNF4A and PPARGC. Further, this suggests that *HNF4A* and *PPARGC* may also be important in HBV regulation by HATs.

## Discussion

HBV replication persists in chronic hepatitis B (CHB) patients, in whom there is an increased risk of developing heptocellular carcinoma (HCC) [13]. Current treatments for CHB include pegylated interferon alfa (PEG-IFNα) and nucleoside or nucleotide analogues (NUCs) [14, 15]. However, PEG-IFNα therapy presents marked side effects [15] and ~40 % sustained virologic response [16]. Furthermore, NUCs drugs cannot completely clear HBV cccDNA from the hepatocyte and HBV mutants appear to be resistant to these drugs during therapy [17]. It is thus necessary to identify alternate targets for novel therapies to suppress HBV replication and to clear HBV [14, 18–20]. The formation of replicative intermediate cccDNA in the nucleus of infected hepatocytes, a process regulated by acetylated histones, is a key event in HBV replication [6, 8]. We thus hypothesize that HATs tightly regulate HBV replication and performed RNAi screening to proof this hypothesis.

The present study showed that HBV replication was regulated tightly by *HAT1* and/or *KAT8*. However, the effects of HAT1 shRNAs and HAT1 inhibitors were not exactly the same, such different effects may be due to different inhibitions at mRNA/protein level by HAT1 shRNAs and HAT1, which will be further clarified in future. Moreover, HBsAg, HBeAg and HBV-DNA were observed as being markedly reduced in HepG2.2.15 cells when *HAT1* or *KAT8* were knocked down. Interestingly, there was minimal up-regulation of HBV-DNA between 72 and 96 h when *HAT1* or *KAT8* was knocked down. In contrast, there was down-regulation of HBsAg with time extension when *KAT8* was knocked down. Indeed, there are inconsistent reports about the association between HBV-DNA and liver histology in the different statuses of HBeAg patients [21]. Moreover, a recent study revealed that large number of virions without HBV-DNA was existed [22], suggesting that HBsAg and/or HBeAg levels probably do not reflect true HBV-DNA level but are instead reflective of the amount of HBV replication. From our qRT-PCR array, we identified a number of HBV-replication-related regulators that were up-regulated by knockdown of each target gene, such as *HNF1A* [23] or *HNF1B*, as well as other transcription factors for HBV replication that were down-regulated, such as PPARGC. These HBV-replication-related factors [24, 25] may preferentially regulate viral RNA synthesis, viral protein translation or viral DNA synthesis during virus lifecycle, thereby leading to different dynamics of HBsAg, HBeAg and HBV-DNA.

It has been demonstrated that *HAT1* plays an important role in controlling inflammatory NF-κB at the transcriptional level by acetylation of the transcriptional regulator PLZF [26]. However, the differential regulation of *HAT1* on HBV-replication-related transcription factors remains to be investigated. *HNF4A* has been demonstrated to promote HBV replication via pgRNA in hepatic and non-hepatic cells [27]. *HNF4A* has been observed as being post-transcriptionally reduced by analogues based on the non-nucleoside natural product helioxanthin, suggesting it as a potential therapeutic target in the management of HBV [28]. In our study, we found that *HAT1* or *KAT8* all modulate *HNF4A* transcription. The peroxisome proliferator-activated receptors (PPARs), namely PPAR-α, PPAR-β, and PPAR-γ, are ligand-activated transcription factors of the nuclear hormone receptor superfamily [24, 25]. These receptors act as lipid metabolism regulators and play key roles in cellular proliferation, differentiation, and apoptosis [29, 30]. In this present study, PPARGC may be demonstrated to be another HBV transcription modulator that is regulated by *HAT1* and *KAT8*. PPARGC synergizes with *HNF4A* to activate downstream genes transcription and HBV replication [31]. These findings suggest that *HNF4A* and *PPARGC* may be critical intermediate modulators that mediate regulation of HBV replication by histone acetyltransferases. In addition to this, *HAT1* and *KAT8* were also observed to differentially modulate expression of HBV transcription regulators, suggesting that they may regulate HBV in different manners.

A more complete understanding of the underlying mechanisms involved in the interaction between HBV and its host factors will be valuable in the identification of new cellular drug targets. The potential use of the novel antiviral targets to inhibit viral replication via interfering virus–host interaction, may have some advantages, such as fewer side effects compared to direct antiviral therapies. In addition, novel therapeutics with a broader spectrum of activity against the various HBV genotypes are necessary to overcome the vast genetic heterogeneity and antiviral drug resistance [32].

## Conclusions

The present study showed that *HAT1* and *KAT8* (human acetyltransferases) play an important role in regulating HBV replication, indicating a potential underlying mechanism by which acetyltransferases influence HBV replication. Our observations may provide novel insights for identifying novel therapeutic targets for treating hepatitis B.

## Methods

### Cell lines and culture

The HBV-replication-stable HepG2.2.15 cell line was maintained in our laboratory, as previously described [9]. HEK293T cells were obtained from the American Type Culture Collection. HepG2.2.15 and HEK293T cells were maintained at 37 °C in DMEM medium, supplemented with 10 % fetal bovine serum (Life Technologies, Carlsbad, CA, USA), in a humidified atmosphere containing 5 % CO_2_. G418 (Life Technologies, Carlsbad, CA, USA) was also added to the medium to a final concentration of 380 μg/mL to maintain the HepG2.2.15 cells [9]. The details of *HAT1* inhibitors were given by the site: http://www.selleck.cn/search.html?searchDTO.searchParam=HAT+inhibitor&sp=HAT%252520inhibitor.

### Preparation of shRNA library and screen

To identify which acetyltransferase genes are involved in HBV replication, RNAi screening was performed, as shown in Fig. 1a. RNAi screening has been widely used in the identification of host factors that regulate infection and replication by pathogens and viruses, including hepatitis C virus (HCV) [33]. shRNAs against 15 human acetyltransferase genes were designed, using an online tool available from the Broad Institute’s RNAi Consortium (TRC) shRNA Library (http://www.broadinstitute.org/rnai/public/), a punchier lentiviral RNAi library that uses multiple distinct shRNA to knockdown most of the known human and mouse genes [34]. Four pairs of oligos were synthesized for each gene and cloned into modified FuGw lentiviral vector, harboring the U6 promoter for shRNA expression and tandem dimer Tomato gene (*Tdtomato*) for infection indication. (All of the oligos and scramble shRNA sequences are listed in Additionalfile 1: Table S1). All of the constructs were verified using colony PCR and using restriction enzyme digestion and subsequent co-transfection with packaging plasmids (Life Technologies, Carlsbad, CA, USA) in HEK293T cells.

HepG2.2.15 cells were infected with pooled lentiviral shRNA library in triplicate plates. The supernatant was collected 72 and 96 h after infection for Quantitative Real-time PCR (qRT-PCR) and HBsAg detection, respectively. RNA was extracted from the cultured cells using Trizol (Life Technologies, Carlsbad, CA, USA) for qRT-PCR. The forward and reverse primers for pgRNA are listed in Additional file 2: Table S2. Cellular RNA was extracted using Trizol (Life Technologies, Carlsbad, CA, USA) and cDNA was prepared from 1 μg total RNA using the RevertAid™ Reverse Transcriptase kit (Thermo Fisher Scientific, Waltham, MA, USA). Amplification was performed as follows: 94 °C for 2 min, then 40 cycles at 95 °C for 15 s, 60 °C for 30 s, 72 °C for 20 s, and 72 °C for 20 s. The primers for *HAT1*, *KAT8* and other genes listed in Additional file 2: Table S2.

### Western blot

The treated cells were lysated with RIPA buffer (Abcam, Cambridge, UK) and collected for western blot. Protein quantification was performed using BCA kit (Abcam, Cambridge, UK). Protein (10 μg) was loaded for electrophoresis and transferred to PVDF membranes. The blots were blocked and labeled with primary antibodies (mouse anti-human HAT1, 1:2000 dilution; Santa Cruz Biotechnology, Dallas, TX, USA); rabbit anti-human KAT8, 1:2000 (Abcam, Cambridge, UK); or mouse anti-human β-actin 1:5000 (Santa Cruz Biotechnology, Dallas, TX, USA) overnight. Membranes were incubated with the secondary antibody (goat anti-rabbit-HRP or goat anti-mouse-HRP, 1:5000 dilution each; Amersham Pharmacia Biotech, Saclay, France). The signal was developed and imaged using ImageQuant™ LAS 4000 (Fujifilm, Tokyo, Japan).

### Enzyme-linked immunosorbent assay (ELISA)

To detect the HBV expression in HepG2.2.15 cells, HBsAg and HBeAg levels in the medium at 72 and 96 h after lentiviral shRNA infection were semi-quantitatively determined using enzyme-linked immunosorbent assay (ELISA) kits (Abbott Diagnostics, Lake Forest, IL, USA) per the manufacturer’s instructions. All experiments were performed in triplicate.

### HBV DNA quantification

To detect the level of HBV replication in HepG2.2.15 cells, supernatant HBV DNA levels in the medium at 72 and 96 h after lentiviral shRNA infection were measured by the Applied Biosystems Real-Time PCR Prism 7500 System (Applied Biosystems, Carlsbad, CA, USA). All experiments were performed in triplicate.

### Cell proliferation assay

Cell proliferation assay was performed in triplicate, using CellTiter 96^®^ AQ_ueous_ One Solution Cell Proliferation Assay kit (Promega, Madison, WI, USA) at 96 h after lentiviral shRNA infection, per the manufacturer’s protocol.

### Statistical analysis

The data are expressed as mean ± SD. One-way ANOVA and two-tailed *Student*’s t-tests were used for Figs. 2, 3 and 4, using GraphPad Prism, Version 6.0 (GraphPad Software, San Diego, CA, USA). *P*-values <0.05 were considered statistically significant.

## Additional files

10.1186/s13578-015-0059-1 Oligos and scramble shRNA sequences.
10.1186/s13578-015-0059-1 Primers for pgRNA and qRT-PCR.

## Abbreviations

*cccDNA*: covalently closed circular DNA
*HAT*: histone acetyltransferase
*HBeAg*: hepatitis B envelop antigen
*HBsAg*: hepatitis B surface antigen
*HBV*: hepatitis B virus
*HCC*: hepatocellular carcinoma
*HDAC*: histone deacetylase
*HNF4A*: hepatocyte nuclear factor-4-α
*PPAR*: peroxisome proliferator-activated receptor
*PPARGC*-*α*: peroxisome proliferator-activated receptor gamma coactivator-1-α
*RC*: relaxed circular DNA
